# Dermatofibrosarcoma Protuberans of the Chest Wall: A Rare Case

**DOI:** 10.7759/cureus.96768

**Published:** 2025-11-13

**Authors:** Varsha R Parupati, Divya Raviprakash, Priya Dharshini R, Leena Joseph, Adikrishnan Swaminathan

**Affiliations:** 1 Dermatology, Venereology and Leprosy, Sri Ramachandra Institute of Higher Education and Research, Chennai, IND; 2 Pathology, Sri Ramachandra Institute of Higher Education and Research, Chennai, IND

**Keywords:** cutaneous sarcoma, dermatofibrosarcoma protruberans, mohs micrographic surgery (mms), skin neoplasms, wide local excision (wle)

## Abstract

Dermatofibrosarcoma protuberans (DFSP) is a rare, slow-growing, low-to-intermediate-grade soft tissue sarcoma, most commonly occurring as a plaque or nodule on the trunk. It can be locally recurrent but very rarely metastasizes. The treatment of choice is wide local excision (WLE). Molecular therapy is useful in those with a pathogenic *COL1A1-PDGFB* gene fusion. We present the case of a 38-year-old male with a slow-growing, mildly pruritic, 10 × 8 cm, skin-colored plaque with a nodular surface on the left side of the anterior chest wall. A skin biopsy showed spindle cells arranged in fascicles and foci with a storiform pattern in the dermis, extending into the subcutis, staining positive for CD34 and negative for S100. Based on these findings, a diagnosis of DFSP was made, and WLE was performed. This case highlights the diagnostic challenges posed by the indolent presentation of DFSP and the importance of early treatment with WLE. Awareness about this rare entity is essential to prevent misdiagnosis and delayed treatment.

## Introduction

Dermatofibrosarcoma protuberans (DFSP) is a rare, slow-growing soft tissue sarcoma of dermal origin. It accounts for around 1.8% of all soft tissue sarcomas [1]. It is considered to be a low-to-intermediate-grade tumor, with a low risk of metastasis, but a high risk of local recurrence. DFSP usually presents as a skin-colored, pink, erythematous, or hyperpigmented plaque or nodule, which slowly enlarges and is most commonly noted on the trunk [2]. Due to its relatively asymptomatic nature, it is often misdiagnosed as a benign lesion. The treatment modality most frequently employed is wide local excision (WLE) or Mohs micrographic surgery [3]. We present the case of a 38-year-old male who presented to our outpatient department with complaints of an occasionally pruritic hyperpigmented indurated plaque on the left side of the chest for five years. A skin biopsy revealed the diagnosis of DFSP. We present this case due to its rarity.

## Case presentation

A 38-year-old male, with no known comorbidities, hailing from South India, presented to our outpatient department with complaints of a raised lesion on his chest for five years. The lesion was insidious in onset and gradually increased in size. A history of occasional mild pruritus was noted. The patient hailed from a rural background and had occasional exposure to river water. There was no history of a sudden increase in the size of the lesion, pain, bleeding, or discharge from the lesion. No similar lesions were noted elsewhere on the body or in other family members. He had a history of application of topical antifungals and emollients on and off for the same.

**Figure 1:**
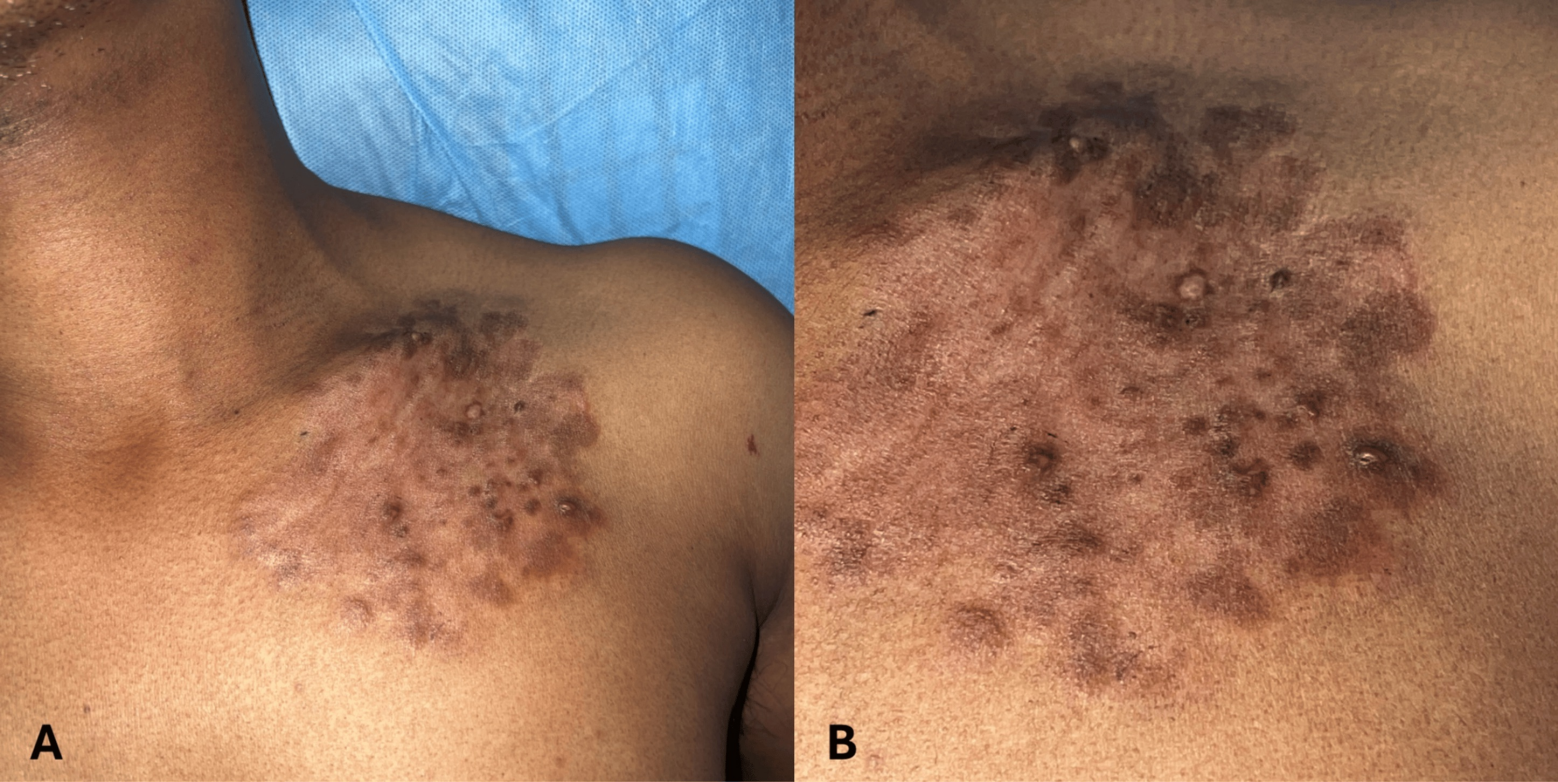
Clinical photograph of the patient at presentation. (A) A 10 × 8 cm well-defined, skin-colored plaque with an irregular nodular surface and areas of hyperpigmentation on the left side of the anterior chest wall. (B) A close-up view of the lesion demonstrating the nodular surface.

On examination, a 10 × 8 cm, well-defined, skin-colored plaque, with an irregular nodular surface and areas of hyperpigmentation, was noted on the left side of the anterior chest wall, extending from 1 cm above the clavicle to 9 cm below the clavicle (Figures 1A, 1B).

There was no local warmth or tenderness. The plaque was fixed to the skin but freely mobile over the underlying structures. There was no regional lymphadenopathy. Systemic examination was within normal limits.

Based on this presentation, a differential diagnosis of Majocchi’s granuloma and *Mycobacterium marinum* infection was primarily considered due to the endemicity of dermatophytes and history of exposure to river water. Other differential diagnoses considered were DFSP and cutaneous sarcoidosis.

**Figure 2:**
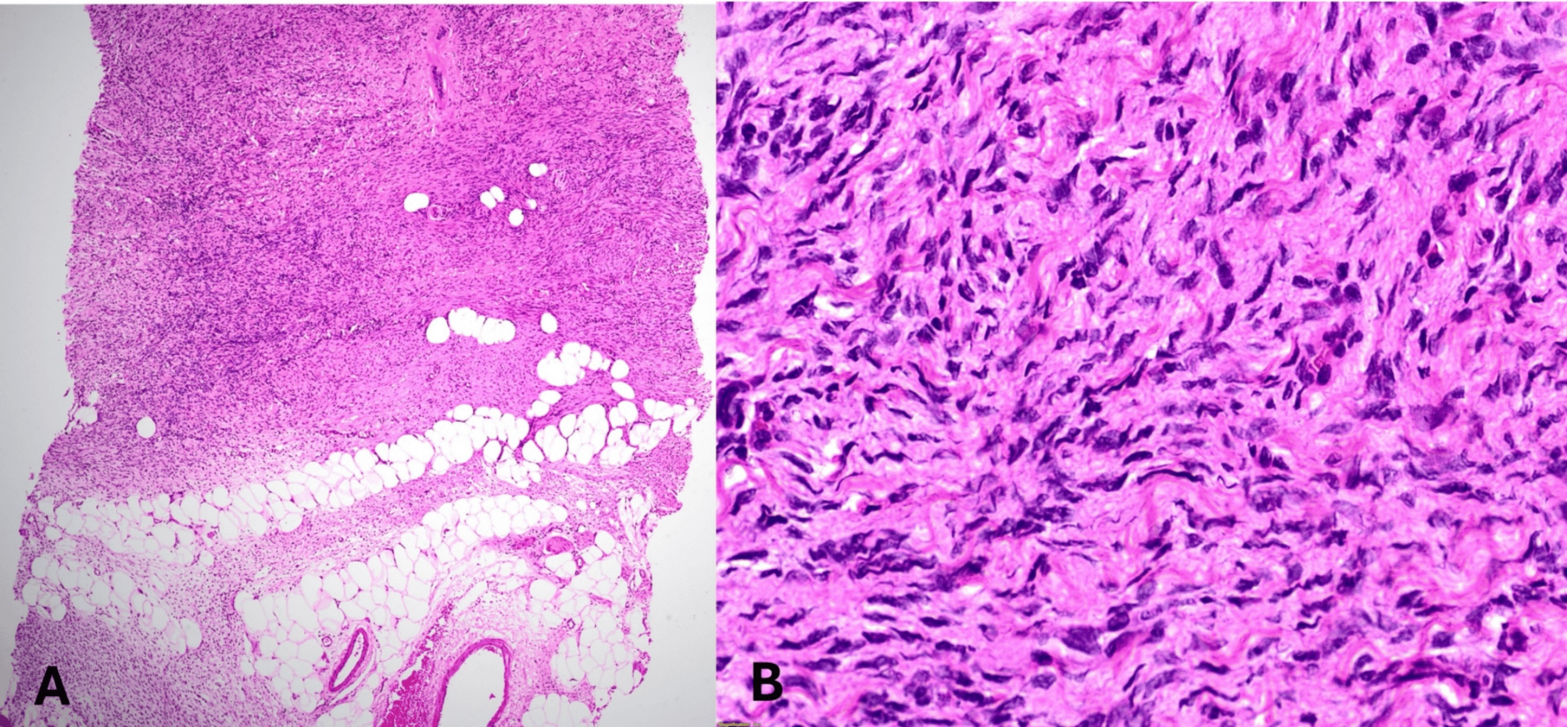
Hematoxylin and eosin staining revealing spindle cells in the dermis arranged in fascicles and foci with a storiform pattern, extending into the subcutaneous fat. (A) 4× magnification. (B) 40× magnification.

A 4-mm skin punch biopsy was obtained from two sites, i.e., from the edge of the plaque and from a nodule within the plaque. Histopathology revealed an unremarkable epidermis and dermis showing spindle cells arranged in fascicles and foci with a storiform pattern, extending into the subcutaneous fat. The spindle cells had elongated, wavy, vesicular nuclei with moderate eosinophilic cytoplasm (Figures 2A, 2B).

**Figure 3:**
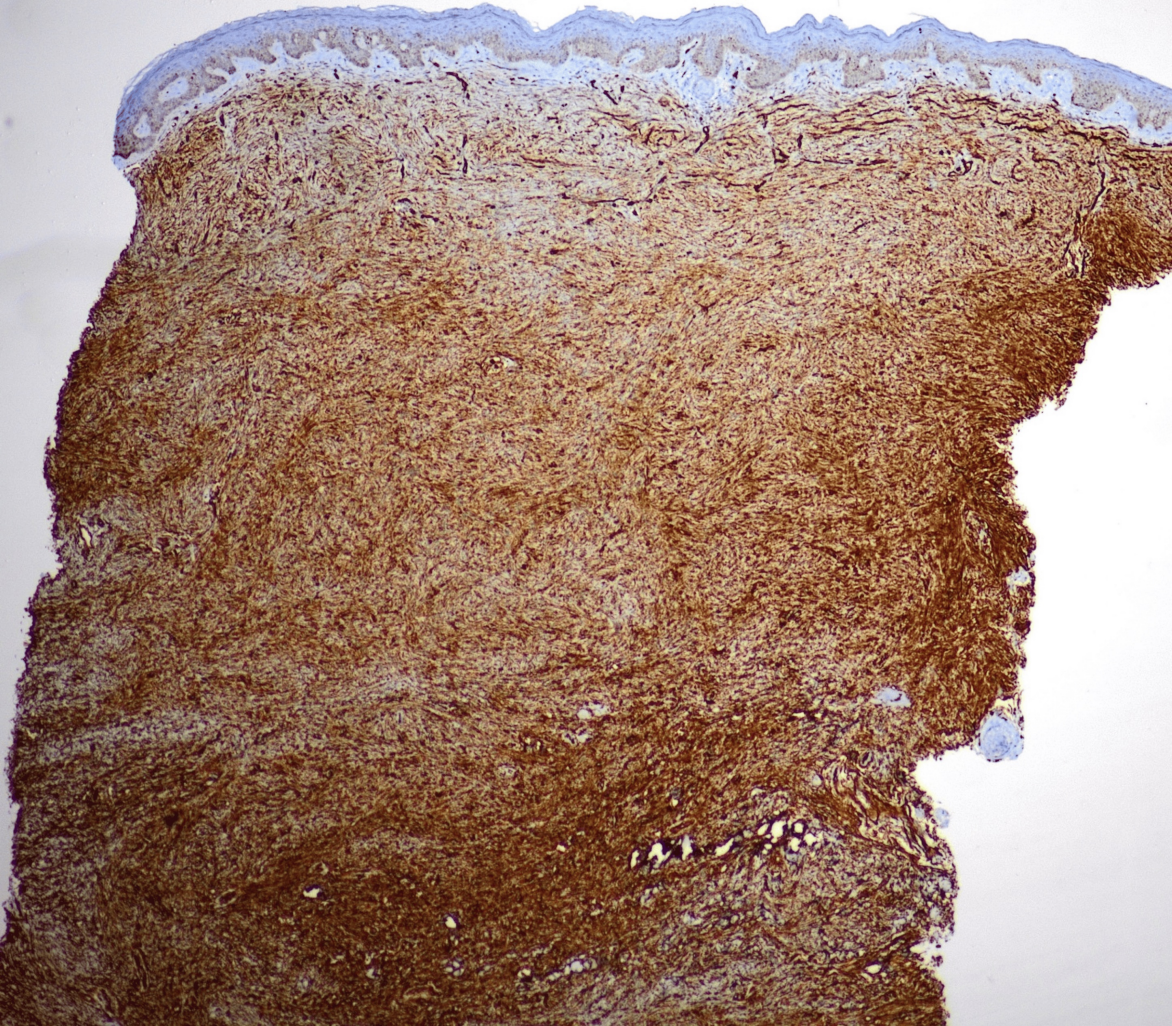
Immunohistochemistry showing cells staining positive for CD34.

**Figure 4:**
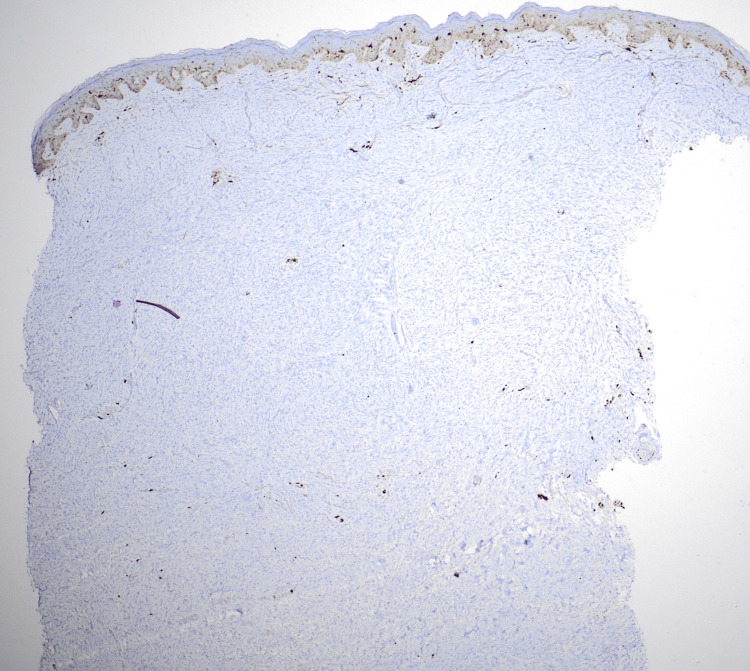
Immunohistochemistry showing cells staining negative for S100.

Immunohistochemistry was advised, and the spindle cells stained positive for CD34 (Figure 3), negative for S100 (Figure 4), and the Ki-67 index was 5%.

Based on this, a diagnosis of DFSP was made. Routine blood investigations were within normal limits. A surgical oncology consult was obtained, and the patient was advised to undergo a WLE of the lesion. The patient underwent WLE with a 3 cm margin up to the fascia with split-thickness skin grafting for reconstruction. Margins were negative for the tumor, and the patient is on regular follow-up, with no evidence of recurrence three months postoperatively.

## Discussion

DFSP is a soft tissue neoplasm, classically categorized as a fibrohistiocytic tumor [2]. It was first described by Taylor in 1980 as a keloidal sarcoma, which Darier and Ferrand later changed to a fibrosarcoma in 1924, and was finally given the name DFSP by Hoffman in 1925 [4]. Although it is the most common cutaneous sarcoma, it is a very rare tumor, accounting for around 0.1% of all malignancies and around 1.8% of soft tissue sarcomas [1,2,5]. It is predominantly encountered in young to middle-aged adults, with a slight female preponderance [2,3].

Almost 90% of cases of DFSP are characterized by the presence of supernumerary ring chromosomes or a specific translocation of chromosomes 17 and 22, which leads to the fusion of the *COL1A1* gene on chromosome 17 with the *PDGFB* gene on chromosome 22. This gene fusion product, *COL1A1-PDGFB*, leads to tumor cell proliferation [3,5]. This is clinically relevant, as patients with this gene rearrangement are ideal candidates for the use of imatinib mesylate, a tyrosine kinase inhibitor.

Clinical presentations of DFSP vary according to the stage at which the patient presents. It is characterized by an initial indolent course, followed by a rapid evolution stage. Patients usually present with a slow-growing, indurated plaque with a nodular surface. The plaque may vary in color from skin-colored to pink-red, violaceous, or hyperpigmented. Nodular irregularity on the surface may occur from months to years after the onset of the plaque. Rarely, it can present directly as a nodule. DFSP lesions are usually fixed to the overlying skin but free from the underlying structures. Invasion beyond the fascia is uncommon but may rarely occur. It is locally recurrent but rarely metastasizes. If metastasis occurs, it is to the lungs, brain, bones, and heart [2]. Other appearances described are morpheaform or sclerodermiform, atrophodermic, and angioma-type. Congenital DFSP is a rare entity; it may present as an erythematous atrophic plaque or an irregular hyperpigmented lesion resembling a nevus. Congenital DFSP is associated with *COL1A1-PDGFB* gene fusion [2,3,5].

On histology, DFSP typically appears as a poorly circumscribed tumor in the dermis, which may extend into deeper structures. It is composed of spindle cells arranged in a storiform pattern, or radially around a central hub, resembling the spokes of a wheel. The cells have large nuclei. Pleomorphism is low, and mitotic figures are infrequent [2]. DFSPs can be differentiated from benign tumors by immunohistochemistry. DFSP stains positive for CD34 and vimentin and negative for S100, factor XIIIa, podoplanin D2-40, stromelysin III, and cathepsin K. This helps differentiate DFSP from benign entities such as dermatofibroma [4]. Other histological variants are pigmented DFSP (Bednar tumor), myxoid DFSP, atrophic DFSP, sclerosing DFSP, giant cell fibroblastoma, granular cell variant, and DFSP-fibrosarcomatous variant [2,4].

Differential diagnoses include dermatofibroma, morphea, keloid, epidermal cysts, morpheaform basal cell carcinoma, amelanotic melanoma, schwannoma, neurofibroma, and cutaneous metastasis. In our case, due to the endemicity of dermatophyte infections, we considered the possibility of Majocchi’s granuloma as well, and due to the history of exposure to river water, *Mycobacterium marinum* infection was also considered.

Imaging, such as MRI, can be done to assess the depth and extent of involvement, which may not be entirely evident on palpation. Other imaging modalities are seldom required due to the rarity of deeper tissue involvement. Extensive evaluation is often unnecessary due to the rarity of lympho-hematogenous dissemination [3].

The mainstay of treatment is WLE with margins of 3-5 cm, including underlying fascial tissue [6]. Mohs micrographic surgery can be employed as a tissue-sparing modality. Recurrence rates are lower with Mohs micrographic surgery compared to WLE [3].

Radiotherapy may be an effective modality, either as a single treatment or as an adjuvant to prevent recurrence after WLE, as DFSP is a radiosensitive malignancy. Chemotherapy has not been proven to be effective, with anecdotal evidence of using low-dose methotrexate with vinblastine and doxorubicin, with no significant response [3].

Targeted molecular therapy with imatinib mesylate is being used for tumors that are not amenable to surgical resection and radiotherapy. Imatinib mesylate is effective in DFSP by inhibiting PDGFR tyrosine kinase, thereby inhibiting cell proliferation and inducing apoptosis of the tumor cells [2]. Molecular therapy is an effective treatment modality in individuals with proven pathogenic *COL1A1-PDGFB* gene fusion.

DFSP has a high rate of recurrence, which is reduced with good surgical margins. Hence, long-term follow-up is prudent to monitor recurrence. Patients who have undergone surgical resection must be followed up every six months for the first three years, followed by annually for life.

## Conclusions

We report a case of DFSP on the anterior chest wall, which was successfully treated with surgical excision and split-thickness skin grafting. DFSP is a rare sarcoma, the diagnosis of which is often delayed due to its indolent course. It can pose a therapeutic challenge due to its recurrence; however, it rarely metastasises. If diagnosed and treated early, the prognosis is excellent. This case report highlights the challenges encountered in the diagnosis of DFSP and emphasizes the need for early diagnosis and treatment.
